# Multidimensional family therapy decreases the rate of externalising behavioural disorder symptoms in cannabis abusing adolescents: outcomes of the INCANT trial

**DOI:** 10.1186/1471-244X-14-26

**Published:** 2014-01-31

**Authors:** Michael P Schaub, Craig E Henderson, Isidore Pelc, Peter Tossmann, Olivier Phan, Vincent Hendriks, Cindy Rowe, Henk Rigter

**Affiliations:** 1 Swiss Research Institute for Public Health and Addiction, associated to University of Zurich, Zurich, Switzerland; 2 Department of Psychology, Sam Houston State University, Huntsville, TX, USA; 3 Department of Psychiatry, CHU Brugmann, Université Libre de Bruxelles, Brussels, Belgium; 4 Delphi-Gesellschaft für Forschung, Berlin, Germany; 5 C.S.P.A. Pierre Nicole, Croix-Rouge Française, Paris, France; 6 Université Paris-Sud et Paris Descartes, Paris, France; 7 Parnassia Addiction Research Centre, The Hague, The Netherlands; 8 Department of Epidemiology and Public Health, University of Miami Miller School of Medicine, Miami, USA; 9 Department of Public Health, Erasmus MC, Rotterdam, The Netherlands; 10 Department of Child and Adolescent Psychiatry, LUMC, Leiden, The Netherlands

**Keywords:** MDFT, Cannabis use disorder, Adolescents, Family therapy, Externalising behavioural symptoms, Family functioning, Europe

## Abstract

**Background:**

US-based trials have shown that Multidimensional Family Therapy (MDFT) not only reduces substance abuse among adolescents, but also decreases mental and behavioural disorder symptoms, most notably externalising symptoms. In the INCANT trial, MDFT decreased the rate of cannabis dependence among Western European youth. We now focus on other INCANT outcomes, i.e., lessening of co-morbidity symptoms and improvement of family functioning.

**Methods:**

INCANT was a randomised controlled trial comparing MDFT with individual therapy (IP) at and across sites in Berlin, Brussels, Geneva, The Hague, and Paris. We recruited 450 boys and girls aged 13 up to 18 years with a cannabis use disorder, and their parent(s), and followed them for 12 months. Mental and behavioural characteristics (classified as 'externalising’ or 'internalising’) and family conflict and cohesion were assessed.

**Results:**

From intake through 12 months, MDFT and IP groups improved on all outcome measures. Models including treatment, site, and referral source showed that MDFT outperformed IP in reducing externalising symptoms.

Adolescents were either self-referred to treatment (mostly on the initiative from people close to the teen) or referred under some measure of coercion by an external authority. These two groups reacted equally well to treatment.

**Conclusions:**

Both MDFT and IP reduced the rate of externalising and internalising symptoms and improved family functioning among adolescents with a cannabis use disorder. MDFT outperformed IP in decreasing the rate of externalising symptoms. Contrary to common beliefs among therapists in parts of Western Europe, the 'coerced’ adolescents did at least as well in treatment as the self-referred adolescents.

MDFT shows promise as a treatment for both substance use disorders and externalising symptoms.

**Trial registration:**

ISRNCT: ISRCTN51014277

## Background

### Why INCANT?

In 1999, the (junior) Ministers of Health of five Western European countries – Belgium, France, Germany, the Netherlands, and Switzerland – concluded that their countries were fighting each other over cannabis policies without sufficient evidence to support any view. Among other things, they decided to fund a transnational trial – named INCANT (INternational CAnnabis Need for Treatment) – to test an outpatient treatment of cannabis use disorder among youth who frequently have co-occurring problems.

The treatment selected was Multidimensional Family Therapy (MDFT), which has been developed from 1985 onwards by Liddle and co-workers mainly at the Center for Treatment Research on Adolescent Drug Abuse (CTRADA), University of Miami Miller School of Medicine. MDFT is a family based outpatient treatment programme for adolescent problem behaviour targeting major domains in the life of an adolescent. The life domains include the youth him- or herself, parent(s), family, friends and peers, school and work, and leisure time. MDFT views family functioning as instrumental in creating new, developmentally adaptive lifestyle alternatives for the adolescent. MDFT has been tested with success in different adolescent populations, doses and treatment delivery settings [1,2].

The history, design, baseline data, and primary (cannabis use related) outcomes of INCANT have been described before [3-5]. Across our research sites in the five Western European countries mentioned, cannabis use disorder was responsive to treatment. Relative to comparison treatment, MDFT was superior in decreasing the prevalence of cannabis dependence, and excelled in reducing the frequency of cannabis consumption in youth with most severe cannabis use [5].

### Secondary outcomes

We now report on other outcomes of INCANT, i.e., effects on co-morbid mental and behavioural symptoms and on family functioning. We call these outcomes 'secondary’ , not because they are of minor importance, but because the primary focus of INCANT was to establish treatment effects on cannabis use variables. In line with previous MDFT research [1,2], we assumed MDFT would be effective in reducing mental and behavioural co-morbidity – internalising and externalising symptoms – in our adolescent cannabis abusing trial participants, while also improving family functioning. Teenagers with internalising symptoms are at increased risk of developing anxiety disorders (symptoms such as feeling nervous, fearful, timid) and mood disorders (symptoms such as feeling lonely, unloved, unhappy, worried, inferior). Externalising symptoms (e.g., arguing, being mean or destructive, getting into fights, stealing, setting fires) are associated for instance with conduct disorder and delinquency [6].

Interactions between substance use, co-morbidity and family factors have been outlined before. In children and adolescents, externalising symptoms and disorders may foreshadow initiation and progression of later cannabis use [7-10], and are associated with an increased risk of later cannabis and other substance dependence [11]. The influence of internalising symptoms on these measures is less clear [12,13]. Family- and peer-related factors may also predict later cannabis use. When family cohesion is low, a teen is more likely to start using cannabis [14], and family conflict may decrease the success of cannabis treatment in adolescents [15]. Poor family functioning is a shared risk factor for cannabis use and mental disorders in adolescents [16]. In a prospective study, externalising symptoms and family dysfunctioning jointly correlated with a higher risk of developing substance use disorders during adolescence [17]. In other words, externalising symptoms (perhaps also internalising symptoms), poor family functioning, and cannabis use are interrelated phenomena.

Thus, a treatment programme like MDFT may reduce cannabis use (problems) along at least two pathways, i.e., through a direct effect on cannabis use, and indirectly by decreasing the impact of co-morbidity and/or family factors. As for the indirect influences, MDFT generally was more effective than cognitive behavioural therapy (CBT) and other active comparison treatments in decreasing both internalising symptoms and externalising symptoms and behaviours [18-20]. If the therapist focuses his or her attention not just on the adolescent but also on the family, this may result in better substance use and externalising/internalising treatment outcomes [21]. We know from recent studies that MDFT reduces cannabis use most strongly in adolescents who have the most severe problems (severity in these studies is defined as heavy drug use or a combination of heavy drug use and more extensive comorbidity including family dysfunction) [5,22,23].

### Objectives

We examined if MDFT had positive effects in adolescents on comorbid mental and behavioural symptoms and on family functioning. We assumed that MDFT would be more effective than the comparison treatment (individual psychotherapy; IP) in reducing the rate of externalising (more so than internalising) symptoms.

## Methods

### Design

INCANT was a phase III(b) randomised controlled effectiveness trial with an open-label, parallel group design, comparing MDFT with individual psychotherapy (IP) across sites in five Western European countries. Study sites started the 24-month recruitment phase between July 2006 and February 2007. Assessments were scheduled at baseline and at 3, 6, 9 and 12 months thereafter. INCANT was approved by an ethical board in each of the countries [3].

### Participants

Eligible participants were teens between the ages of 13 and 18 years from both genders with a cannabis use disorder (dependence or abuse) established as having been present within the year prior to baseline. At least one parent had to take part in the treatment.

Adolescents were ineligible if they suffered from a current mental disorder or condition requiring inpatient treatment (e.g., psychosis, advanced eating disorder, suicidal ideation), or had a substance use disorder requiring maintenance treatment with methadone or buprenorphine. Cases were excluded if the adolescent and/or parent were unable to speak and read the local language.

We consecutively recruited 450 cases/families, with 60 to 120 cases/families per site depending on the local budget available [5].

The referral source for the adolescents varied [4]. We distinguished two classes of referral. Self-Determined (SD) referral applied to adolescents who took the initiative to contact the treatment site on the advice (with no obligation) of relatives, friends, acquaintances, school, and occasionally on their own initiative. Externally Coerced (EC) referral applied to adolescents who felt they could not refuse treatment out of fear of sanctions, such as being kicked out of something (school, services, or programmes), being placed out of home, or being detained or otherwise being sanctioned by Justice authorities. This included referral by youth probation officers or appointed family guardians, public prosecutors, or court.

### Study sites; randomisation

Sites were selected from addiction, youth and forensic care centres upon nomination by Government officials, after site visits by study staff, and on the basis of performance in a pilot study [3]. The sites were (a) the outpatient cannabis clinic of the department of psychiatry of Brugmann University Hospital in Brussels, (b) Therapieladen in Berlin, (c) Centre Emergence in Paris with suburban CEDAT (Conseils Aide et Action contre le Toximanie) sub-sites in Mantes la Jolie and St Germain en Laye, (d) the twin sites of Parnassia Brijder (addiction care) and De Jutters (forensic care) in The Hague, and (e) Phénix in Geneva. All sites were youth oriented [5].

Concealed randomisation took place per site, using three stratification variables (gender, age and frequency of cannabis consumption) [3], right after the case had been found eligible at the baseline assessment. For each stratum, the database computer generated 50 independent randomisations [5].

The database automatically assigned a code to each new case entered by a research assessor and informed her about the allocated treatment, independently from any trial staff [3,5].

### Therapists and interventions

Different therapists from the same site delivered either MDFT or IP. They were similar in age, gender, education and experience [24]. Training procedures for MDFT therapists have been described before [24], and included a two weeks course in using the treatment manual, site visits, and active supervision during the study regarding session planning, case assessment and developing the treatment plan. Moreover, a sample of recordings of sessions were independently reviewed on measures of treatment adherence and competence [24]. For IP, procedures for intervision and supervision were already in place, and where these were found wanting, they were upgraded to the level required for the study.

An average of two MDFT sessions per week was prescribed – in roughly equal proportion to be held with the adolescent, parent, and family (adolescent and parent together), respectively. Sessions could take place at the office of the therapist, the family’s home, or any other location. IP was to last as long as MDFT (6 months), but with fewer sessions per week. Details on the actual treatment dose received have been reported before [24].

IP was individual counselling of the adolescent, which was treatment as usual across sites. IP was not standardised across sites; this would have required a revolution in deeply rooted treatment practices and would have aroused opposition from professional societies and other stakeholders – a challenge well beyond our capabilities. IP varied from full CBT in The Hague [23] and Brussels to more elective approaches in the other countries. MDFT and IP were comparable in terms of motivational interviewing, intervision (peer consulting), administrative and referral procedures, and drug education [5,24].

### Outcome measures

Cannabis use was assessed with the Timeline Follow-Back method (TLFB), a well-validated self-report method to record frequency of cannabis use as adapted for adolescents [25]. The TLFB registers daily cannabis use for the 90 days preceding the assessment, using a calendar and other memory prompts.

Also, urine samples were taken but these data were incomplete in Switzerland, where many clinicians regard urine testing as breaching the personal integrity of clients. This was already expected, and accepted as unchangeable, at the time the trial was designed. The laboratory results are not reported here.

Adolescents’ internalising and externalising symptoms were recorded with the Youth Self Report (YSR). This instrument is reliable and valid across a variety of studies, populations, and languages (including Dutch, German and French) [26,27]. For the parents’ view of the internalising and externalising symptoms of their children, the parent version of the YSR, called the CBCL (Child Behaviour Checklist), was used [28]. The data used were from the parent most directly involved in raising the adolescent as established at baseline or from the parent volunteering to contribute to all follow-up assessments. The YSR and CBCL were scheduled at baseline and at six and twelve months.

We used the Anxiety/Depression, Withdrawn, and Somatic complaints sub-scales of the YSR and CBCL as components of the Internalising symptoms scale of the YSR and CBCL, which has been validated [29]. A high score on this scale means that the adolescent (or in the CBCL, the parent) reported a high rate of internalising symptoms. For externalising symptoms, we used another validated scale, based on the sub-scales Delinquency and Aggressive behaviour [29]. There is only one question on substance use in the YSR and CBCL among the 30 YSR externalising symptoms items, so overlap between our primary and secondary outcome measures was negligible.

Family conflict and cohesion were assessed with the respective sub-scales from the Family Environment Scale (FES), a widely used and well-validated self-report measure [30], which was completed by the teen. The FES was delivered at baseline and at six, nine and twelve months.

With a single exception, YSR, CBCL and FES analyses reported here are across-site. Information on outcomes per site has been published before [31].

### Analyses

We followed an intent-to-treat approach, with change in the YSR, CBCL, and FES outcomes (assessed at intake, 6, 9 [FES only], and 12 months) analysed using latent growth curve modelling (LGC; [32]) conducted with the software Mplus ([33], version 6). Both intercept and slope were modelled separately for each outcome. We included site and referral source (self-directed or externally coerced referral) as covariates, based on INCANT’s baseline findings [4], along with treatment condition. Site by treatment interactions were not statistically significant and therefore were omitted from the final models. Missing data were handled with full information maximum likelihood estimation, under the missing at random assumption [34].

A statistically significant (p < 0.05) slope parameter, as tested by the pseudo-z test, indicated the intervention was effective. For each slope intercept, we provide the 95% confidence interval (CI). In conditional models, MDFT was coded as 0 and IP as 1; thus, positive slope coefficients associated with treatment would indicate greater decreases in internalising and externalising problems for youth receiving MDFT. In addition, the magnitude of the coefficient indicated deviation from the mean slope associated with MDFT [32].

For comparing sites on continuous data we used analysis of variance (ANOVA).

## Results

Sites varied on some baseline measures, as reported before [4,31]. As for measures to be used in the analysis of secondary outcomes, the between-site difference in YSR self-report of externalizing symptoms was most notable (ANOVA; *F* [4, 425] = 6.4, *p* < 0.001). Youth in Germany reported higher levels of externalizing symptoms than youth in France and the Netherlands. Differences on other baseline measures were negligible. Within sites, there were no baseline discrepancies between the two treatment arms.

Study dropout was low with, across-site, 90% of cases completing the 12-month follow-up in the MDFT group and 89% in the IP group [5].

### LGC modelling

Table 1 shows the means and standard deviations for each secondary outcome measure at each assessment wave (baseline, 6 and 12 months for YSR and CBCL; and baseline, 6, 9 and 12 months for FES). From baseline through 12 months, across sites and treatments and across the assessment points mentioned for the various questionnaires, adolescents improved on all secondary outcome variables: YSR-internalising slope = -0.80, 95% CI = -1.02 to -0.58, pseudo-z = -6.74, p < 0.001; YSR-externalising slope = -2.46, 95% CI = -2.86 to -2.06, pseudo-z = -12.35, p < 0.001; CBCL-internalising slope = -1.60, 95% CI = -1.86 to -1.34, pseudo-z = -12.07, p < 0.001; CBCL-externalising slope = -4.50, 95% CI = -5.08 to -3.92, pseudo-z = -15.56, p < 0.001; FES-cohesion slope = 0.06, 95% CI = 0.04 to 0.08, pseudo-z = 12.00, p < 0.001; FES-conflict slope = 0.06, 95% CI = 0.04 to 0.08, pseudo-z = 12.47, p < 0.001 (higher FES-conflict scores reflect less conflict; therefore, a positive slope value means less conflict). Table 2 lists the model parameters.

**Table 1:** Observed sample sizes, means, and standard deviations for self- and parent-reported internalising and externalising symptoms and for family functioning

**Outcome measure**	**Intake M (*SD*)**	**6 Month M (*SD*)**	**9 Month M ( *SD *)**	**12 Month M ( *SD *)**
*YSR Internalising*	*n = 430*	*n = 346*		*n = 382*
MDFT	14.59 (9.75)	10.96 (7.77)	N/A	10.82 (8.87)
IP	14.60 (9.56)	11.99 (8.71)	N/A	11.76 (9.23)
*YSR Externalising*	*n = 430*	*n = 346*		*n = 382*
MDFT	21.57 (9.22)	17.08 (8.61)	N/A	15.38 (9.07)
IP	19.73 (8.32)	17.12 (9.21)	N/A	15.86 (8.80)
*CBCL Internalising*	*n = 433*	*n = 341*		*n = 363*
MDFT	20.14 (10.32)	14.81 (9.75)	N/A	13.08 (9.79)
IP	21.12 (11.18)	16.12 (10.90)	N/A	13.96 (9.16)
*CBCL Externalising*	*n = 434*	*n = 341*		*n = 364*
MDFT	26.25 (12.04)	18.44 (10.68)	N/A	16.34 (11.15)
IP	23.84 (11.51)	18.76 (12.39)	N/A	15.35 (9.80)
*FES Conflict*^a^	*n = 429*	*n = 345*	*n = 308*	*n = 357*
MDFT	0.43 (0.21)	0.59 (0.22)	0.65 (0.15)	0.59 (0.20)
IP	0.45 (0.21)	0.62 (0.20)	0.67 (0.13)	0.63 (0.21)
*FES Cohesion*	*n = 429*	*n = 345*	*n = 308*	*n = 357*
MDFT	0.63 (0.30)	0.83 (0.27)	0.80 (0.17)	0.85 (0.25)
IP	0.60 (0.29)	0.79 (0.27)	0.77 (0.18)	0.81 (0.26)

*Note.* Total sample was n = 450 adolescents. The table presents the number of cases (n) per outcome variable and assessment point on which the figures shown are based. YSR = Youth Self-Report, MDFT = Multidimensional Family Therapy, IP = Individual psychotherapy, N/A = Not applicable, CBCL = Child Behaviour Checklist (as completed by 1 parent), FES = Family Environment Scale.

^a^Higher scores reflect less conflict.

**Table 2:** Means and standard errors for growth factors and phase differences on outcomes

	**Intercept**		**Slope**	
	***M***	***SE***	***M***	***SE***
Parent-reported externalising				
Growth factor mean	24.59^***^	0.57	-4.50^***^	0.29
Treatment comparison	-1.79	1.12	0.57	0.56
Site comparison	-0.03	0.53	-0.32	0.28
Referral source comparison	2.44	1.31	-1.54^*^	0.67
Parent-reported internalising				
Growth factor mean	20.17^***^	0.53	-3.48^**^	0.25
Treatment comparison	0.95	1.01	0.03	0.51
Site comparison	-0.07	0.52	-0.13	0.24
Referral source comparison	-0.72	1.25	-0.47	0.42
Youth-reported externalising				
Growth factor mean	20.34^***^	0.42	-2.46^***^	0.11
Treatment comparison	-1.52	0.84	1.00^*^	0.41
Site comparison	-0.03	0.39	-0.11	0.20
Referral source comparison	0.39	0.98	0.31	0.49
Youth-reported internalising				
Growth factor mean	14.07^***^	0.45	-1.61^*^	0.20
Treatment comparison	0.19	0.89	0.52	0.44
Site comparison	-0.15	0.37	-0.05	0.20
Referral source comparison	0.07	1.03	0.63	0.51
Family cohesion				
Growth factor mean	0.64^***^	0.02	0.06^***^	0.01
Treatment comparison	<0.01	0.04	-0.02	0.01
Site comparison	0.01	0.02	0.01	0.01
Referral source comparison	<0.01	0.04	-0.01	0.02
Family conflict				
Growth factor mean	0.49^***^	0.02	0.06^***^	0.01
Treatment comparison	0.04	0.03	<0.01	0.01
Site comparison	<0.01	0.01	<0.01	0.01
Referral source comparison	0.01	0.03	<0.01	0.01

^*^p < .05, ^**^p < .01, ^***^p < .001.

In most of these changes for the better, and at the across-site overall level, MDFT and IP did equally well. Running models including treatment condition pointed to one measure where MDFT outperformed IP, i.e., the decrease in self-reported (YSR) externalising problems (slope coefficient on treatment = 1.00, 95% CI = 0.18 to 1.82, pseudo z = 2.45, p = 0.01, d = 0.26). This was not reflected in the parents’ reports (CBCL): slope coefficient on treatment = 0.57, 95% CI = -0.55 to 1.69, pseudo z = 1.02, p = 0.31.

### The influence of referral

External coercion did not harm treatment outcome. Across sites and treatments, SD and EC adolescents improved to the same degree on secondary outcomes. EC adolescents even showed greater improvement on the YSR externalising symptoms measure (slope coefficient on referral source = -1.54, 95% CI = -2.88 to -0.20, pseudo z = -2.29, p = 0.02; d = 0.30).

## Discussion and conclusions

MDFT and IP reduced youth-reported internalising and externalising disorder symptoms and increased family functioning (more cohesion, less conflict). This is not just a reflection of 'passage of time’ as MDFT outperformed IP in decreasing the rate of externalising, but not internalising symptoms.

MDFT is often regarded as a substance abuse treatment programme, for historic reasons (funding sources and initial research interests) and achieved outcomes, but the approach is also valuable for treating adolescent externalising symptoms and delinquency. In teens, substance use frequently occurs along with a constellation of other problems [35,36]. In earlier trials [18-20] and the present one, MDFT reduced the rate of externalising symptoms, more so than active comparison treatments. Lowering the level of externalising symptoms may help a youth to refrain from criminal offenses [35,36]. The effect of treatment on externalising symptoms merits further study in relation to the distal goal of reducing criminal offenses.

When we mounted INCANT, clinicians from especially Switzerland criticised our choice to also recruit adolescents referred by Justice authorities. There is a strong belief among some groups of therapists in Western Europe that coercing a youth into treatment will harm the chances of the therapist to establish a therapeutic alliance with the adolescent, and for the treatment to be successful. Our data did contradict this notion. Adolescents coerced into treatment accepted therapy and stayed in therapy as long as other teens [24]. Our findings show that these coerced adolescents did respond to treatment even better (on the externalising symptoms measure) than other teens.

How to interpret this finding? There is older literature on the effect of coerced *treatment* among detainees, but reports on coerced *referral* in *outpatient* settings are scarce and based on studies of limited quality. Nevertheless, it would appear that coercion does not hurt and may even improve outcomes [37].

Substance abuse treatment may lower the rate of internalising symptoms in adolescents, but the evidence is inconsistent [1,5,7,8]. Our data confirm that treatment can reduce the number of internalising symptoms. MDFT did not differ from IP here.

We used well-tested and reliable self-report questionnaires [25-30]. YSR outcomes did not (fully) match CBCL outcomes, as has been noted before [38]. Discrepancies in YSR versus CBCL scores (different views of the adolescent and the parent as to symptoms present) may signify higher levels of psychopathology among the adolescents [39]. Both YSR and CBCL are valid as measured against clinically established diagnoses [40]. Yet, adolescents and their parents may have different perceptions of what is going on in the life of the teen. There are speculations about this discrepancy in views, but a convincing explanation is still wanting.

MDFT views the family as the prime environment to facilitate and strengthen treatment effects. Family conflict will make it harder to positively respond to treatment [1,2]. Our data confirm the importance of family conflict. Teens from conflict-prone families benefitted more from MDFT than from IP in reducing days of cannabis use. There was no effect on the family conflict measure itself. Possibly, our methods were not precise enough to measure changes in family functioning. The FES questionnaire asks about the whole family, and specifically about the teen - parent relationship.

Our study had limitations. One theory holds that systems therapies such as MDFT increase parents’ competence, thus improving parental behaviour, and subsequently improving the behaviour of their child. Our study did not include measures of parental behaviour, so we cannot say if this chain of events did occur. However, a detailed process study of another systems therapy (MST) found evidence in support of the importance of these intermediate steps in the link between treatment and outcomes [41].

We recruited adolescents from widely varying backgrounds and different countries, excluding few, to match the population accepted for treatment in daily practice, which enhances the external validity of the study findings. Accordingly, the results of INCANT have high external validity. In other words, they are generalisable to regular treatment settings.

Our results confirm that MDFT is an effective programme for treating substance abusing adolescents, especially adolescents with high rates of externalising symptoms. One could wonder how the effect of MDFT progresses. Does this treatment programme first weaken externalising and perhaps other co-morbid symptoms and, through this pathway, then continue to reduce substance use and substance abuse? The available evidence suggests that MDFT may do both: influencing substance use (and other problem behaviour) through a direct effect on this behaviour, and diminishing substance use (problems) indirectly, by affecting moderator variables such as externalising symptoms. And the reverse, i.e., direct and indirect influences of substance (ab)use on externalising symptoms developing into for instance delinquency, is also true. The evidence for the existence of both pathways has been brought together in the Host-Provocation theory [42]. Advanced statistical models confirm that substance use and co-morbidity factors may interact in intricate ways to elicit and strengthen unwanted behaviours in adolescents [43].

## Abbreviations

CBCL: Child behaviour checklist; CBT: Cognitive behavioural therapy; FES: Family environment Scale; INCANT: International cannabis need of treatment study; IP: Individual psychotherapy of various therapeutic orientations common at the INCANT treatment sites; LGC modelling: Latent growth curve modelling; M: Mean; MDFT: Multidimensional family therapy; Mdn: Median; SD: Standard deviation; TLFB: Timeline follow-back; YSR: Youth self report.

## Competing interests

CR trains teams of therapists in MDFT as a consultant. HR has established MDFT training programmes in Europe. All other authors declare that they have no competing interests.

## Authors’ contributions

All authors were substantially involved in the implementation of the study. MS was the principal investigator (PI) for INCANT in Switzerland and drafted the manuscript. CEH carried out the statistical analyses and he and HR helped to complete the manuscript. IP, PT, OP, and VH were responsible for data collection as PIs in their respective countries. HR and CR designed the overall study, which was being coordinated by HR. All authors read and approved the final manuscript.

## Pre-publication history

The pre-publication history for this paper can be accessed here:

http://www.biomedcentral.com/1471-244X/14/26/prepub
